# *catena*-Poly[[[2-({6-[(pyrimidin-2-ylsulfanyl-κ*S*)meth­yl]pyridin-2-yl-κ*N*}methyl­sulfan­yl)pyrimidine]­copper(I)]-μ-thio­cyanato-κ^2^ *N*:*S*]

**DOI:** 10.1107/S160053681201063X

**Published:** 2012-03-17

**Authors:** Rong Peng, Seik Weng Ng

**Affiliations:** a Department of Chemistry, Shantou University, Shantou 515063, Guangdong, People’s Republic of China; b Department of Chemistry, University of Malaya, 50603 Kuala Lumpur, Malaysia; c Chemistry Department, King Abdulaziz University, PO Box 80203 Jeddah, Saudi Arabia

## Abstract

The *N*-heterocyclic ligand in the title compound, [Cu(NCS)(C_15_H_13_N_5_S_2_)]_*n*_, coordinates to the Cu^I^ atom through its pyridine N-donor site, and adjacent metal atoms are bridged by the thio­cyanate ion, forming a helical chain along the *b* axis. The geometry of the metal atom is tetra­hedral owing to a somewhat long intra­molecular Cu—S inter­action of 2.5621 (9) Å.

## Related literature

For the synthesis of the *N*-heterocyle and its copper(I) adducts, see: Peng *et al.* (2006 ▶).
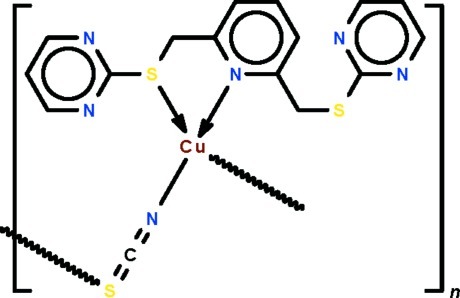


## Experimental

### 

#### Crystal data

[Cu(NCS)(C_15_H_13_N_5_S_2_)]
*M* *_r_* = 449.04
Monoclinic, 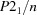

*a* = 11.1706 (8) Å
*b* = 8.6735 (6) Å
*c* = 19.0956 (14) Å
β = 100.978 (1)°
*V* = 1816.3 (2) Å^3^
*Z* = 4
Mo *K*α radiation
μ = 1.56 mm^−1^
*T* = 293 K
0.16 × 0.11 × 0.06 mm

#### Data collection

Bruker SMART APEX CCD diffractometer
Absorption correction: multi-scan (*SADABS*; Sheldrick, 1996 ▶) *T*_min_ = 0.703, *T*_max_ = 1.000
10799 measured reflections
4077 independent reflections
3022 reflections with *I* > 2σ(*I*)
*R*_int_ = 0.028

#### Refinement

*R*[*F* ^2^ > 2σ(*F* ^2^)] = 0.046
*wR*(*F* ^2^) = 0.125
*S* = 1.04
4077 reflections
235 parameters
H-atom parameters constrained
Δρ_max_ = 1.18 e Å^−3^
Δρ_min_ = −0.51 e Å^−3^

### 

Data collection: *SMART* (Bruker, 2002 ▶); cell refinement: *SAINT* (Bruker, 2002 ▶); data reduction: *SAINT*; program(s) used to solve structure: *SHELXS97* (Sheldrick, 2008 ▶); program(s) used to refine structure: *SHELXL97* (Sheldrick, 2008 ▶); molecular graphics: *X-SEED* (Barbour, 2001 ▶); software used to prepare material for publication: *publCIF* (Westrip, 2010 ▶).

## Supplementary Material

Crystal structure: contains datablock(s) global, I. DOI: 10.1107/S160053681201063X/zs2186sup1.cif

Structure factors: contains datablock(s) I. DOI: 10.1107/S160053681201063X/zs2186Isup2.hkl

Additional supplementary materials:  crystallographic information; 3D view; checkCIF report

## Figures and Tables

**Fig. 1.**
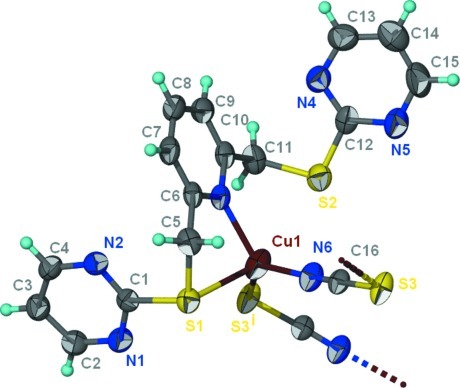
Thermal ellipsoid plot (Barbour, 2001) of a portion of the polymeric chain structure of [Cu(NCS)(C16H13N5S2)]n at the 50% probability level. Hydrogen atoms are drawn as spheres of arbitrary radius.

**Fig. 2.**
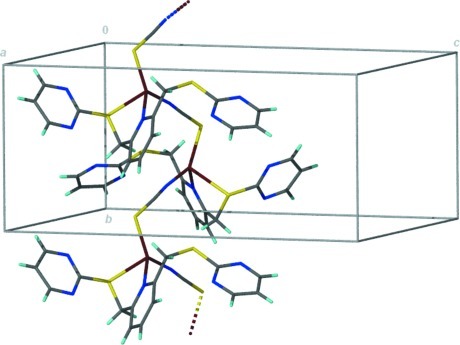
The chain structure of the title compound, extending along the b-axis of the unit cell.

## supplementary crystallographic information

### Comment

We have previously reported the crystal structures of the copper(I) bromide and iodide adducts of 2,6-bis(2-pyrimidinesulfanylmethyl)pyridine. The ligand is a flexible thioether than can coordinate through the nitrogen and sulfur sites (Peng *et al.*, 2006). In the present copper(I) thiocyanate adduct (Scheme I), the *N*-heterocyclic ligand coordinates to the Cu^I^ atom through its pyridyl *N*-donor site (Fig. 1). Adjacent metal atoms are bridged by the thiocyanate ion to form a helical chain running along the *b*-axis of the monoclinic unit cell (Fig. 2). The geometry of the metal atom is a tetrahedron owing to a somewhat long intramolecular sulfur–copper interaction of 2.5621 (9) Å.

### Experimental

The ligand was synthesized as described by Peng *et al.* (2006). Copper(I) thiocyanate (0.012 g, 1 mmol), 2,6-bis(2-pyrimidinesulfanylmethyl)pyridine (0.032 g, 0.1 mmol) and acetonitrile (4 ml) were placed in a 13-ml, Teflon-line, stainless-steel Parr bomb. This was heated at 373 K for 48 h, and then cooled at 3 K a minute. The solution was filtered and the solvent allowed to evaporate over two weeks to give brown prisms.

### Refinement

Carbon-bound H-atoms were placed in calculated positions (C—H = 0.95–0.97 Å) and were included in the refinement in the riding model approximation, with *U*_iso_(H) set to 1.2*U*_eq_(C). The final difference Fourier map had a peak (1.179 eÅ^-3^in the vicinity of Cu1.

### Crystal data

**Table:**

[Cu(NCS)(C_15_H_13_N_5_S_2_)]	*F*(000) = 912
*M**_r_* = 449.04	*D*_x_ = 1.642 Mg m^−^^3^
Monoclinic, *P*2_1_/*n*	Mo *K*α radiation, λ = 0.71073 Å
Hall symbol: -P 2yn	Cell parameters from 2743 reflections
*a* = 11.1706 (8) Å	θ = 2.6–25.0°
*b* = 8.6735 (6) Å	µ = 1.56 mm^−^^1^
*c* = 19.0956 (14) Å	*T* = 293 K
β = 100.978 (1)°	Prism, brown
*V* = 1816.3 (2) Å^3^	0.16 × 0.11 × 0.06 mm
*Z* = 4	

### Data collection

**Table:**

Bruker SMART APEX CCD diffractometer	4077 independent reflections
Radiation source: fine-focus sealed tube	3022 reflections with *I* > 2σ(*I*)
Graphite monochromator	*R*_int_ = 0.028
ω scans	θ_max_ = 27.5°, θ_min_ = 2.2°
Absorption correction: multi-scan (*SADABS*; Sheldrick, 1996)	*h* = −13→14
*T*_min_ = 0.703, *T*_max_ = 1.000	*k* = −11→4
10799 measured reflections	*l* = −24→23

### Refinement

**Table:**

Refinement on *F*^2^	Primary atom site location: structure-invariant direct methods
Least-squares matrix: full	Secondary atom site location: difference Fourier map
*R*[*F*^2^ > 2σ(*F*^2^)] = 0.046	Hydrogen site location: inferred from neighbouring sites
*wR*(*F*^2^) = 0.125	H-atom parameters constrained
*S* = 1.04	*w* = 1/[σ^2^(*F*_o_^2^) + (0.0592*P*)^2^ + 0.7767*P*] where *P* = (*F*_o_^2^ + 2*F*_c_^2^)/3
4077 reflections	(Δ/σ)_max_ = 0.001
235 parameters	Δρ_max_ = 1.18 e Å^−^^3^
0 restraints	Δρ_min_ = −0.51 e Å^−^^3^

### Fractional atomic coordinates and isotropic or equivalent isotropic displacement parameters (Å^2^)

**Table:**

	*x*	*y*	*z*	*U*_iso_*/*U*_eq_	
Cu1	0.40542 (4)	0.22472 (5)	0.23431 (3)	0.06021 (17)	
S1	0.39013 (7)	0.38008 (9)	0.11883 (4)	0.0492 (2)	
S2	0.51157 (8)	0.14604 (12)	0.39459 (5)	0.0604 (2)	
S3	0.05933 (8)	0.47654 (10)	0.29022 (5)	0.0583 (2)	
N1	0.4820 (3)	0.2133 (3)	0.02977 (15)	0.0547 (7)	
N2	0.6034 (2)	0.4274 (3)	0.07782 (14)	0.0522 (6)	
N3	0.5545 (2)	0.3694 (3)	0.26678 (12)	0.0404 (5)	
N4	0.6536 (3)	0.3205 (4)	0.49195 (15)	0.0615 (8)	
N5	0.4632 (3)	0.2354 (4)	0.51544 (18)	0.0740 (9)	
N6	0.2579 (3)	0.3150 (3)	0.25756 (16)	0.0560 (7)	
C1	0.5066 (3)	0.3375 (4)	0.07088 (15)	0.0425 (6)	
C2	0.5642 (3)	0.1795 (4)	−0.00998 (18)	0.0573 (8)	
H2	0.5505	0.0949	−0.0404	0.069*	
C3	0.6680 (3)	0.2642 (4)	−0.00794 (18)	0.0565 (9)	
H3	0.7248	0.2391	−0.0360	0.068*	
C4	0.6842 (3)	0.3875 (4)	0.03729 (18)	0.0578 (9)	
H4	0.7544	0.4465	0.0401	0.069*	
C5	0.4517 (3)	0.5411 (4)	0.17298 (17)	0.0508 (7)	
H5A	0.4792	0.6173	0.1424	0.061*	
H5B	0.3865	0.5875	0.1928	0.061*	
C6	0.5557 (3)	0.5052 (3)	0.23334 (15)	0.0428 (7)	
C7	0.6469 (3)	0.6129 (4)	0.25452 (18)	0.0542 (8)	
H7	0.6454	0.7066	0.2307	0.065*	
C8	0.7392 (3)	0.5806 (5)	0.31075 (19)	0.0611 (9)	
H8	0.8015	0.6513	0.3254	0.073*	
C9	0.7378 (3)	0.4420 (4)	0.34490 (18)	0.0549 (8)	
H9	0.7993	0.4179	0.3834	0.066*	
C10	0.6455 (3)	0.3388 (4)	0.32216 (16)	0.0463 (7)	
C11	0.6440 (3)	0.1836 (4)	0.35597 (19)	0.0582 (8)	
H11A	0.7163	0.1736	0.3930	0.070*	
H11B	0.6487	0.1055	0.3202	0.070*	
C12	0.5486 (3)	0.2465 (4)	0.47565 (18)	0.0514 (8)	
C13	0.6736 (4)	0.3913 (5)	0.5549 (2)	0.0738 (11)	
H13	0.7461	0.4454	0.5686	0.089*	
C14	0.5936 (4)	0.3884 (6)	0.5997 (2)	0.0797 (12)	
H14	0.6095	0.4388	0.6435	0.096*	
C15	0.4898 (4)	0.3090 (7)	0.5780 (2)	0.0903 (15)	
H15	0.4334	0.3051	0.6080	0.108*	
C16	0.1773 (3)	0.3838 (3)	0.26969 (16)	0.0443 (7)	

### Atomic displacement parameters (Å^2^)

**Table:**

	*U*^11^	*U*^22^	*U*^33^	*U*^12^	*U*^13^	*U*^23^
Cu1	0.0529 (3)	0.0354 (2)	0.0982 (4)	0.00026 (17)	0.0294 (2)	−0.0022 (2)
S1	0.0522 (4)	0.0427 (4)	0.0549 (4)	0.0001 (3)	0.0157 (3)	0.0011 (3)
S2	0.0644 (5)	0.0590 (6)	0.0588 (5)	−0.0066 (4)	0.0142 (4)	0.0121 (4)
S3	0.0532 (5)	0.0343 (4)	0.0968 (7)	0.0037 (3)	0.0376 (4)	0.0064 (4)
N1	0.0595 (17)	0.0439 (16)	0.0633 (16)	−0.0106 (13)	0.0185 (13)	−0.0113 (13)
N2	0.0567 (16)	0.0472 (16)	0.0533 (15)	−0.0099 (13)	0.0123 (12)	−0.0070 (12)
N3	0.0459 (13)	0.0321 (13)	0.0478 (13)	0.0040 (10)	0.0206 (11)	0.0004 (10)
N4	0.0522 (17)	0.075 (2)	0.0563 (17)	−0.0033 (15)	0.0080 (13)	0.0150 (15)
N5	0.0558 (18)	0.095 (3)	0.076 (2)	−0.0009 (17)	0.0254 (15)	0.0030 (19)
N6	0.0511 (15)	0.0437 (15)	0.0782 (18)	0.0038 (13)	0.0252 (14)	0.0003 (14)
C1	0.0507 (16)	0.0363 (15)	0.0401 (14)	0.0009 (13)	0.0080 (12)	0.0061 (12)
C2	0.066 (2)	0.0468 (18)	0.0612 (19)	−0.0061 (16)	0.0175 (17)	−0.0147 (16)
C3	0.057 (2)	0.058 (2)	0.0585 (19)	0.0017 (16)	0.0199 (16)	−0.0053 (16)
C4	0.0519 (18)	0.061 (2)	0.063 (2)	−0.0144 (16)	0.0168 (16)	−0.0090 (17)
C5	0.066 (2)	0.0342 (16)	0.0550 (17)	0.0072 (15)	0.0190 (15)	0.0018 (14)
C6	0.0543 (16)	0.0332 (15)	0.0477 (15)	0.0029 (13)	0.0268 (13)	−0.0035 (12)
C7	0.068 (2)	0.0411 (17)	0.0615 (19)	−0.0099 (16)	0.0331 (17)	−0.0036 (15)
C8	0.059 (2)	0.063 (2)	0.067 (2)	−0.0173 (18)	0.0274 (18)	−0.0150 (18)
C9	0.0469 (17)	0.065 (2)	0.0551 (18)	0.0031 (16)	0.0159 (14)	−0.0075 (17)
C10	0.0495 (17)	0.0447 (17)	0.0506 (17)	0.0086 (14)	0.0244 (14)	0.0015 (14)
C11	0.063 (2)	0.057 (2)	0.0587 (19)	0.0121 (17)	0.0203 (16)	0.0087 (17)
C12	0.0459 (17)	0.0512 (19)	0.0583 (18)	0.0115 (14)	0.0133 (14)	0.0189 (15)
C13	0.075 (3)	0.076 (3)	0.064 (2)	−0.008 (2)	−0.001 (2)	0.010 (2)
C14	0.091 (3)	0.081 (3)	0.067 (2)	0.017 (3)	0.014 (2)	−0.004 (2)
C15	0.078 (3)	0.128 (4)	0.076 (3)	0.016 (3)	0.040 (2)	−0.006 (3)
C16	0.0457 (16)	0.0334 (15)	0.0566 (17)	−0.0040 (13)	0.0166 (13)	0.0032 (13)

### Geometric parameters (Å, º)

**Table:**

Cu1—N6	1.951 (3)	C2—H2	0.9300
Cu1—N3	2.083 (2)	C3—C4	1.364 (5)
Cu1—S3^i^	2.2536 (9)	C3—H3	0.9300
Cu1—S1	2.5621 (9)	C4—H4	0.9300
S1—C1	1.767 (3)	C5—C6	1.505 (4)
S1—C5	1.794 (3)	C5—H5A	0.9700
S2—C12	1.756 (4)	C5—H5B	0.9700
S2—C11	1.804 (3)	C6—C7	1.385 (4)
S3—C16	1.653 (3)	C7—C8	1.369 (5)
S3—Cu1^ii^	2.2536 (9)	C7—H7	0.9300
N1—C1	1.331 (4)	C8—C9	1.369 (5)
N1—C2	1.331 (4)	C8—H8	0.9300
N2—C1	1.319 (4)	C9—C10	1.372 (5)
N2—C4	1.341 (4)	C9—H9	0.9300
N3—C6	1.341 (4)	C10—C11	1.494 (5)
N3—C10	1.346 (4)	C11—H11A	0.9700
N4—C12	1.322 (4)	C11—H11B	0.9700
N4—C13	1.329 (5)	C13—C14	1.351 (6)
N5—C12	1.331 (4)	C13—H13	0.9300
N5—C15	1.337 (6)	C14—C15	1.344 (7)
N6—C16	1.140 (4)	C14—H14	0.9300
C2—C3	1.367 (5)	C15—H15	0.9300
			
N6—Cu1—N3	110.66 (11)	H5A—C5—H5B	107.4
N6—Cu1—S3^i^	128.26 (9)	N3—C6—C7	121.8 (3)
N3—Cu1—S3^i^	118.42 (7)	N3—C6—C5	118.0 (3)
N6—Cu1—S1	93.67 (9)	C7—C6—C5	120.1 (3)
N3—Cu1—S1	81.83 (7)	C8—C7—C6	119.6 (3)
S3^i^—Cu1—S1	108.00 (4)	C8—C7—H7	120.2
C1—S1—C5	102.84 (15)	C6—C7—H7	120.2
C1—S1—Cu1	113.65 (10)	C7—C8—C9	118.6 (3)
C5—S1—Cu1	87.58 (10)	C7—C8—H8	120.7
C12—S2—C11	101.37 (17)	C9—C8—H8	120.7
C16—S3—Cu1^ii^	103.70 (10)	C8—C9—C10	119.9 (3)
C1—N1—C2	115.3 (3)	C8—C9—H9	120.1
C1—N2—C4	114.7 (3)	C10—C9—H9	120.1
C6—N3—C10	118.2 (3)	N3—C10—C9	122.0 (3)
C6—N3—Cu1	117.9 (2)	N3—C10—C11	116.6 (3)
C10—N3—Cu1	123.8 (2)	C9—C10—C11	121.4 (3)
C12—N4—C13	115.2 (3)	C10—C11—S2	114.6 (2)
C12—N5—C15	114.5 (3)	C10—C11—H11A	108.6
C16—N6—Cu1	172.1 (3)	S2—C11—H11A	108.6
N2—C1—N1	127.7 (3)	C10—C11—H11B	108.6
N2—C1—S1	119.7 (2)	S2—C11—H11B	108.6
N1—C1—S1	112.6 (2)	H11A—C11—H11B	107.6
N1—C2—C3	122.7 (3)	N4—C12—N5	127.0 (3)
N1—C2—H2	118.7	N4—C12—S2	119.9 (2)
C3—C2—H2	118.7	N5—C12—S2	113.2 (3)
C4—C3—C2	116.6 (3)	N4—C13—C14	123.2 (4)
C4—C3—H3	121.7	N4—C13—H13	118.4
C2—C3—H3	121.7	C14—C13—H13	118.4
N2—C4—C3	123.0 (3)	C15—C14—C13	116.7 (4)
N2—C4—H4	118.5	C15—C14—H14	121.7
C3—C4—H4	118.5	C13—C14—H14	121.7
C6—C5—S1	115.8 (2)	N5—C15—C14	123.5 (4)
C6—C5—H5A	108.3	N5—C15—H15	118.2
S1—C5—H5A	108.3	C14—C15—H15	118.2
C6—C5—H5B	108.3	N6—C16—S3	177.0 (3)
S1—C5—H5B	108.3		
			
N6—Cu1—S1—C1	−178.20 (14)	C10—N3—C6—C5	−178.1 (2)
N3—Cu1—S1—C1	71.44 (13)	Cu1—N3—C6—C5	−2.2 (3)
S3^i^—Cu1—S1—C1	−45.88 (12)	S1—C5—C6—N3	−35.0 (3)
N6—Cu1—S1—C5	78.83 (14)	S1—C5—C6—C7	147.2 (2)
N3—Cu1—S1—C5	−31.53 (12)	N3—C6—C7—C8	0.5 (4)
S3^i^—Cu1—S1—C5	−148.84 (11)	C5—C6—C7—C8	178.2 (3)
N6—Cu1—N3—C6	−67.0 (2)	C6—C7—C8—C9	−0.5 (5)
S3^i^—Cu1—N3—C6	129.96 (18)	C7—C8—C9—C10	0.4 (5)
S1—Cu1—N3—C6	23.86 (18)	C6—N3—C10—C9	0.2 (4)
N6—Cu1—N3—C10	108.6 (2)	Cu1—N3—C10—C9	−175.4 (2)
S3^i^—Cu1—N3—C10	−54.4 (2)	C6—N3—C10—C11	−177.3 (2)
S1—Cu1—N3—C10	−160.5 (2)	Cu1—N3—C10—C11	7.0 (3)
C4—N2—C1—N1	1.0 (5)	C8—C9—C10—N3	−0.3 (5)
C4—N2—C1—S1	−177.9 (2)	C8—C9—C10—C11	177.2 (3)
C2—N1—C1—N2	−1.8 (5)	N3—C10—C11—S2	−61.7 (3)
C2—N1—C1—S1	177.2 (3)	C9—C10—C11—S2	120.8 (3)
C5—S1—C1—N2	−4.7 (3)	C12—S2—C11—C10	−79.1 (3)
Cu1—S1—C1—N2	−97.7 (2)	C13—N4—C12—N5	−0.5 (6)
C5—S1—C1—N1	176.2 (2)	C13—N4—C12—S2	−179.2 (3)
Cu1—S1—C1—N1	83.2 (2)	C15—N5—C12—N4	0.3 (6)
C1—N1—C2—C3	1.2 (5)	C15—N5—C12—S2	179.1 (3)
N1—C2—C3—C4	−0.2 (6)	C11—S2—C12—N4	−0.6 (3)
C1—N2—C4—C3	0.3 (5)	C11—S2—C12—N5	−179.5 (3)
C2—C3—C4—N2	−0.7 (5)	C12—N4—C13—C14	0.3 (6)
C1—S1—C5—C6	−71.3 (2)	N4—C13—C14—C15	0.0 (7)
Cu1—S1—C5—C6	42.4 (2)	C12—N5—C15—C14	0.1 (7)
C10—N3—C6—C7	−0.3 (4)	C13—C14—C15—N5	−0.2 (8)
Cu1—N3—C6—C7	175.6 (2)		

Symmetry codes: (i) −*x*+1/2, *y*−1/2, −*z*+1/2; (ii) −*x*+1/2, *y*+1/2, −*z*+1/2.
